# A Pilot Test of an AI Voice-Driven Simulation With Feedback for Medical Students to Practice Discussing Diagnostic Mammogram Results With Patients

**DOI:** 10.7759/cureus.95606

**Published:** 2025-10-28

**Authors:** Warren S Comulada, Patricia A Ganz, Yue Ming Huang, Dan Weisman, Alanna Sugarman, Daniel Lee, Saachi Shah, Lillian Gelberg

**Affiliations:** 1 Health Policy and Management, University of California Los Angeles (UCLA) Fielding School of Public Health, Los Angeles, USA; 2 Psychiatry and Biobehavioral Sciences, University of California Los Angeles (UCLA) David Geffen School of Medicine, Los Angeles, USA; 3 Oncology, University of California Los Angeles (UCLA) David Geffen School of Medicine, Los Angeles, USA; 4 Medical Education and Simulation, University of California Los Angeles (UCLA) Simulation Center, Los Angeles, USA; 5 Anesthesiology and Perioperative Medicine, University of California Los Angeles (UCLA) David Geffen School of Medicine, Los Angeles, USA; 6 Medicine, University of California Los Angeles (UCLA) David Geffen School of Medicine, Los Angeles, USA; 7 Family Medicine, University of California Los Angeles (UCLA) David Geffen School of Medicine, Los Angeles, USA; 8 Psychology, University of California Los Angeles (UCLA), Los Angeles, USA

**Keywords:** breast biopsy, gpt-4, large language model (llm), mammography, screen-based simulation, teaching communication skills, virtual standardized patient

## Abstract

Introduction

Effective communication when delivering sensitive results or bad news to patients is necessary for medical professionals, warranting greater attention in medical education. The opportunity to practice communication skills with human standardized patients has limitations, including not being readily available, prompting researchers to develop virtual patients (VPs) to supplement training with a focus on routine tasks. This study capitalized on artificial intelligence (AI) advances to develop a VP for more complex dialogs where learners practice discussing uncertain screening results with patients.

Methods

We conducted a single-arm pilot study with 10 medical students to evaluate the feasibility and acceptability of a simulated telephone call between a physician (learner) and a VP to discuss the patient’s abnormal mammogram results that require breast biopsy examination. Objectives culminate in the VP agreeing to schedule a biopsy. Afterward, an AI agent displays feedback for the learner.

Results

Study completion demonstrated feasibility, with quantitative measures indicating acceptability. The average System Usability Scale score of 91 was high (range, 78-100). For Technology Acceptance Model questions, most students “agreed” or “strongly agreed” that VP responses “seemed reasonable”, AI agent-generated feedback was “helpful”, and VP training would increase their “confidence communicating with patients”. Qualitative feedback solidified these findings, highlighting the simulation’s realism, detailed feedback, and perceived effectiveness in improving communication skills.

Conclusion

The study recommends further evaluation for including VPs in medical education, especially to help learners practice difficult conversations with patients. Future developments will expand the scenario and evaluate communication skills outcomes while continuing to refine the VP and feedback.

## Introduction

Effective communication between providers and their patients drives cancer prevention and management efforts [1]. Clinicians need to deliver test results in a timely manner to optimize cancer diagnosis and treatment [2]. Delivery may entail breaking uncertain or bad news, like a cancer diagnosis, which is a difficult task that can psychologically impact physicians delivering the news, patients receiving the news, and their family members [3-6]. In recognition of its importance, clinical educators are devoting more time in medical school curricula to communication skills training [7]. Medical students simulate clinical patient encounters by communicating with standardized patients (SPs).

Despite its importance, communication skills training is limited by competing clinical demands, the availability of SPs, and their ability to portray raw emotions across the spectrum of patient encounters [8-11]. Another challenge is the evaluation of training programs to improve and maintain their quality, which requires additional educator effort and the need to standardize evaluations across evaluators [9,10]. Training is focused on the most common types of patient encounters that are the easiest to implement and evaluate, such as physical examinations, with less attention to the delivery of bad news regarding serious illnesses [7,11].

Computer-simulated or virtual patients (VPs) are a tantalizing solution to scale SP programs beyond limited in-person engagements, but have historically been hindered by high costs and logistical challenges [12,13]. The advent of the third Generative Pre-trained Transformer (GPT-3) in 2020 and other large language models (LLMs) reduced developmental and deployment barriers, and rekindled efforts among researchers and clinical educators to develop VPs. Since then, researchers have made advances in developing GPT-enabled VPs, but the focus has still been on routine provider-patient conversations in alignment with their human counterparts, e.g., history taking [14,15], diagnosis [12,16-17], disease management [12], and prenatal counseling [18].

We address communication skills training gaps through a GPT-4-enabled VP we developed for medical students to practice less routine and potentially more difficult conversations with patients. The scenario is a simulated telephone call between the learner, acting as a primary care physician (PCP), and their patient that is simulated by the VP. The patient has received screening and diagnostic mammograms that have produced abnormal results and require further examination through a breast biopsy. The patient has questions about their results they want to discuss with their PCP before further follow-up.

This article reports the results of a feasibility and acceptability study that pilot tested the VP with medical students. The primary objective was to determine whether the VP-enabled simulation could provide a realistic, emotionally engaging, and technically reliable learning experience that complements existing SP training at our institution’s medical school. Specifically, we sought to examine how well learners could engage in a simulated telephone conversation with the VP, receive AI-generated feedback, and reflect on their communication approach. The study introduces four key innovations: (1) a communication scenario involving diagnostic uncertainty and the possibility of cancer to enhance emotional complexity; (2) real-time verbal learner-VP interactions enabled by GPT-4 with integrated speech-to-text and text-to-speech capabilities, similar to the study by Maquilón et al. [19], as opposed to most VP-based simulations using text-only communication; (3) an AI-driven feedback agent that evaluates learner performance and displays written feedback to the learner on a computer screen, as seen in studies by Holderried et al. [14] and Webb [20], in contrast to simulations that automate feedback using point-based systems; and (4) deployment of the VP-enabled simulation through an institutional learning management system (LMS) to enable scalable access. Study objectives and innovations collectively guided the assessment of the VP-enabled simulation’s potential for integration into medical education.

## Materials and methods

Overview

We report the results of a single-arm pilot study that took place from July to August 2024. The study evaluated the feasibility, acceptability, and effectiveness of a communication skills training module that incorporated a VP to help medical students practice discussing abnormal results of a diagnostic mammogram with a patient. We recruited 10 medical students from the University of California, Los Angeles (UCLA) David Geffen School of Medicine who had completed their first year of education. We obtained ethical approval for all study procedures from the UCLA Institutional Review Board (23-001385). All participants provided consent to participate.

Simulation module and VP

**Figure 1:**
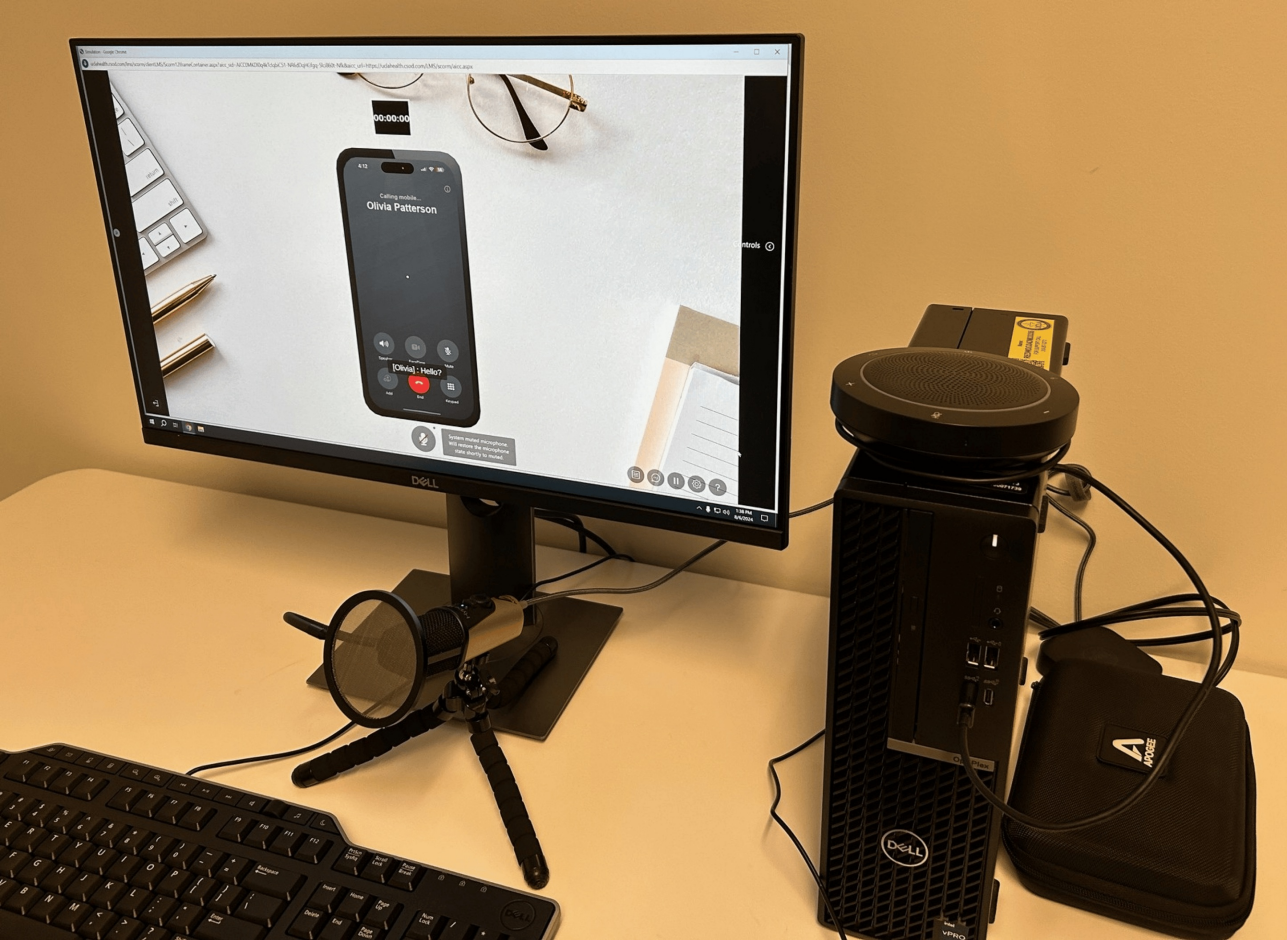
Desktop computer and microphone study participants used to interact with the virtual patient. The computer screen shows a picture of a cell phone to simulate a telephone call with the virtual patient, named Olivia Patterson. Image credit: Warren S. Comulada (first author)

We used HyperSkill (SimInsights, Irvine, USA), a virtual simulation authoring software, to simulate a phone call between the learner and a VP to discuss the VP’s abnormal mammogram results. Based on PCP input, we simulated a phone call and not an office visit to mimic the mode of communication they reported using to discuss abnormal results from a mammogram with patients [21]. The application is displayed on a computer screen and features visuals of a cell phone on a desk, as shown in Figure 1. We iteratively developed a prompt to provide input to the GPT-powered VP and achieved a desired level of role-playing consistency for learner-VP conversations. This prompt included a patient backstory along with behavioral constraints about how to interact with the learner.

We leveraged an AI-powered agent (i.e., a clinician coach) to provide written feedback/evaluation to the learner after they finished their conversation with the VP. To do this, we developed a second prompt for the clinician coach to analyze the learner’s interaction and provide specific feedback about the learner’s ability to adhere to the Setting, Perception, Invitation, Knowledge, Empathy, and Strategy and Summary (SPIKES) protocol for breaking bad news [22,23], and persuade the VP to proceed with the recommended next steps (scheduling a breast biopsy). These prompts were iteratively designed and play-tested to ensure that both the VP and simulated clinician coach would respond appropriately to a broad range of possible learner dialog (see [21] for further details).

Participants and procedures

We recruited UCLA medical students aged 18 years and older through an email invitation sent to first-year medical students. The email contained a link to an electronic screener, consent, and Qualtrics survey that participants filled out prior to the study visit (see Appendix A for survey questions). Eligible individuals were enrolled in their first year of medical school at UCLA and at least 18 years old. Conversely, individuals not enrolled in their first year of medical school at UCLA or younger than 18 years of age were ineligible. We deployed the simulation through the Cornerstone OnDemand LMS (Santa Monica, USA), an online human resources management and training system used at our university. After enrollment, we directed participants to log into Cornerstone and complete an online didactic module before their in-person study visit to pilot test the simulation (Appendix B). The didactic module provided general information about difficult patient conversations and the SPIKES protocol for breaking bad news. We designed the didactic module to take no more than 30 minutes to complete and gave participants access to the module 48 hours before their scheduled study visit.

Participants pilot tested the simulation at the UCLA Simulation Center in a private room. They first completed a pre-simulation module that provided information about the VP’s mammogram results, guidance on how to interpret the mammogram results, and the recommended next steps. It also provided learners with AI-generated content disclaimers, outlining potential biases, inaccuracies, and other associated risks and drawbacks of using AI-generated responses and feedback. They then accessed the simulation and began a simulated phone call to discuss abnormal mammogram results. They were seated in front of a desktop computer and spoke into a computer microphone to interact with the VP. After completing the simulation and receiving automated feedback on their performance, students completed a post-pilot Qualtrics survey evaluating their impressions of the training (Appendix A).

Measures

Background Characteristics

We collected participants’ demographic data through a Qualtrics survey at enrollment, including age, race/ethnicity, and sex assigned at birth. Participants were asked whether they had received formal training in breaking bad news to patients and about their computer gaming experience, categorized as little to no experience, casual, or hardcore.

Feasibility

We evaluated feasibility by the ability of participants to complete the pilot study.

Acceptability

Qualitative and quantitative survey questions we administered through the post-pilot Qualtrics survey informed acceptability. We collected qualitative data through three pairs of open-ended questions assessing what participants liked or found “most useful” and what they disliked or found “least useful” regarding the online didactic and pre-simulation modules, the interaction with the VP, and the automated feedback they received. We then queried participants on “other topics or scenarios” they would find appropriate for this type of simulation that would help them gain experience delivering bad or difficult news to patients. We also asked participants if there were other formats, besides a virtual simulation, they would find appropriate to gain experience delivering difficult news to patients and concluded the survey by asking participants to share additional comments.

We administered the 10-item System Usability Scale (SUS) in its original form that queried the usability of a “system” except to query about the usability of a “virtual training module” [24,25]. The SUS uses a five-point Likert scale with response categories ranging from “I strongly disagree” (1) to “I strongly agree” (5) and produces a summary score (range, 0-100). Scores of 68 or higher indicate good to excellent usability and acceptability. We administered 14 questions adapted from the Technology Acceptance Model (TAM) and used the same five-point Likert scale we used for SUS questions [26-29]. Since the original TAM questions queried the acceptance of “electronic mail”, we revised questions to evaluate various aspects of the virtual training module, such as the clarity of instructions, ease of navigation, and technical functionality. Participants also assessed the realism of the simulated interactions, the usefulness of the feedback, and whether the module increased their confidence and communication skills in delivering difficult news. The questions further explored whether the module enhanced learning, enjoyment, and engagement. Participants were asked if they would recommend the module to others and if they would like to see similar virtual simulations for other types of patient interactions.

Effectiveness

We preliminarily evaluated effectiveness through a question asking participants how prepared they felt to deliver bad news in the pre- and post-pilot surveys. Response options comprised a five-point Likert scale categorized as “Very unprepared”, “Unprepared”, “Neither prepared or unprepared”, “Prepared”, and “Very prepared”.

Analysis

We calculated summary statistics for sociodemographic characteristics and quantitative measures like the SUS. Co-authors reviewed and summarized responses to open-ended questions by categorizing them into likes/dislikes about each component of the training module (didactic/pre-simulation, simulation, and feedback), other simulation topics proposed by participants, and general comments. They finalized categories after reaching consensus with the research team.

## Results

Table 1 presents demographic and background characteristics for the 10 pilot study participants. The median age of the participants was 25 years (range, 23-30 years old). The sample was fairly balanced between female and male participants based on sex assigned at birth. Half of the participants reported Asian race; the remaining half of the sample was diverse across racial/ethnic categories. All but one participant reported having received formal training in breaking bad news to patients. The sample was balanced between participants with little to no gaming experience and casual gamers.

**Table 1:** Demographic and background characteristics for 10 participants in the pilot study ^*^Current gender identity matched sex at birth for nine participants; one participant did not report their current gender identity.

Characteristics	n	%
Median age in years (range)	25 (23-30)	
Sex assigned at birth^*^		
Female	6	60%
Male	4	40%
Race/ethnicity		
Asian	5	50%
Black/African American	1	10%
Hispanic/Latino	1	10%
Native Hawaiian/Pacific Islander	1	10%
White	1	10%
Did not specify	1	10%
Received formal training in how to break bad news to patients		
Yes	9	90%
No	1	10%
Gaming experience		
Little to no experience	5	50%
Casual gamer	5	50%

Feasibility was indicated by all participants completing the didactic, pre-simulation, and virtual simulation modules.

Table 2 summarizes qualitative data from open-ended post-pilot survey questions and illustrative paraphrased quotations from participants. Participant responses indicated high levels of acceptability across all three sections of the training module: the online didactic and pre-simulation modules, the VP they interacted with as part of the simulation during the in-person visit, and the automated feedback the simulation provided during the in-person visit. Participants appreciated reviewing the SPIKES protocol during the didactic module, the realism of their interaction with the VP, the emotional connection they felt during the simulated telephone call, and the detailed feedback they received at the end of the simulation. Participants were favorable to the way they received feedback; the AI-agent-generated feedback was displayed alongside the transcript of their dialog with the VP. One participant commented how this facilitated detailed feedback to help improve “specific phrases or parts of the interaction” that would be less practical with human evaluators relying on human memory.

**Table 2:** Categories and illustrative paraphrased quotations from medical student responses to open-ended questions about their likes and dislikes for components of the communication skills module (didactic/pre-simulation, simulated conversation with the virtual patient, and feedback), suggestions for future (other) topics and formats. VP: virtual patient; SPIKES protocol: Setting, Perception, Invitation, Knowledge, Empathy, and Strategy and Summary protocol ^*^Indicates medical students in order of participation.

Categories	Paraphrased quotations	Medical student number*
Introduction (didactic/pre-simulation) likes		
Clear objectives	“I liked having the objectives of the simulated phone call clearly stated as part of the introduction”.	3
Review of protocol for breaking bad news (i.e., SPIKES)	“The presentation of the SPIKES protocol with graphics was a helpful refresher”.	4
Introduction dislikes		
Unclear instructions for interaction with VP	“The introduction should have included instructions on how to interact with the virtual patient, e.g., indicating that the phone call microphone would start off muted”.	3
Simulation likes		
Realism of VP interaction	“I liked the authenticity of the virtual patient's voice, emotions, and responses”.	10
Emotional connection	“I experienced similar emotional responses when talking to the virtual simulation as in clinical encounters with real patients”.	7
Simulation dislikes		
AI limitations	“I was uncertain about how many sentences I could speak to the virtual patient. As a result, my conversation may have been disjointed”.	2
	“If I did not address part of the question from the virtual patient in that moment, I might not have a chance to address it later in the conversation”.	8
Usability	“I would sometimes forget to unmute the microphone or to not mute the microphone after speaking”.	4
Feedback likes		
Level of detail	“The feedback was thorough. Standardized patients are not typically able to remember the conversation verbatim, making it difficult to receive detailed feedback about how we can improve specific parts of the conversation”.	1
Transcript review	“The quotes were really good to highlight what we said well, but also sample lines that we could've said to make it even better”.	5
Feedback dislike: Limited feedback	“I would have liked more feedback on areas to improve”.	9
Future topics		
Mental health	Simulations that emulate virtual patients with mental health disorders, experiences of abuse, and other conditions patients present with during their clinical visits	1
Different difficulty levels	Simulations with tiers of difficulty for delivering bad news	8
Working with translators	Simulations where learners work with a translator to communicate with a virtual patient	8
Future format: Integration with human standardized patient training	In-person training with standardized patients is crucial for medical training, but this type of training is helpful to prepare for in-person training or as additional training.	8

Participants emphasized the value of VP training to complement in-person SP training. For example, one participant wanted the VP training to be “implemented into FOP (Foundations of Practice)”. FOP is a course for first-year medical school students at our institution where they practice their interpersonal communication skills through simulated patient encounters with SPs. There was strong support for expanding the use of virtual simulations to cover additional difficult conversations that physicians have with patients about other types of cancers (e.g., “pancreatic cancer”), chronic conditions (e.g., “diabetes”), “mental health disorders”, “substance use disorder”, and traumatic events. Participants also proposed simulations to include physician conversations with “family members (i.e., teen with parents)”.

Critiques were minor, mostly requesting further instruction on how to interact with the VP in the introductory material and highlighting AI limitations during VP interactions. Two participants requested further instruction on how to interact with the VP through the microphone during the introduction, and a third participant indicated uncertainty as to when to mute their microphone after speaking. One participant noted that they were unsure how many sentences they could speak to the VP, which may have resulted in a “disjointed” conversation. Another participant felt they had to completely address VP questions each time they responded to the VP, unlike interactions with real patients where they could return to finish addressing questions later in the conversation. Despite positive indications about the VP’s realism, one participant indicated that the VP’s voice “sounded a bit too AI”. Participants wanted more critical feedback about their communication skills. Aside from critiquing the simulation itself, one participant felt the topic area was beyond their level of training.

Responses to quantitative measures mirrored high levels of acceptability from the qualitative data. The average SUS score was 91 (range, 78-100). For TAM question responses in Table 3, all but one participant agreed or strongly agreed that the “simulated (AI generated) patient responses seemed reasonable.” All participants agreed or strongly agreed that the “feedback I received at the end of the simulation was helpful” and that “using the virtual training module would increase my confidence communicating with patients”. Most participants expressed interest in VP training in the future and would recommend it to others.

**Table 3:** Number and percentage of participants indicating they “strongly agree” in responding to statements for survey items derived from the Technology Acceptance Model. Remaining responses were “I agree” or “I do not agree or disagree”.

Statements	n	%
Instructions on how to use the virtual training module were clear and understandable.	10	100%
The user interface for the virtual training module was easy to navigate.	9	90%
I did not encounter tech issues while using the virtual training module.	9	90%
The simulated phone call felt real to me.	5	50%
The simulated (AI generated) patient responses seemed reasonable to me.	5	50%
I found the feedback I received at the end of the simulation to be helpful.	9	90%
Using the virtual training module would increase my confidence communicating with patients.	9	90%
Using the virtual training module would improve my learning and performance delivering difficult news.	9	90%
Using the virtual training would make it easier for me to understand communication concepts.	8	80%
I enjoyed using the virtual training module.	9	90%
Using a virtual training module makes learning more interesting.	7	70%
I would like to use the virtual training module in the future if I have the opportunity.	9	90%
I would recommend the training module to others to help them practice difficult conversations with patients.	9	90%
I would like to see more of these types of virtual simulations for different types of patient encounters.	9	90%

**Figure 2:**
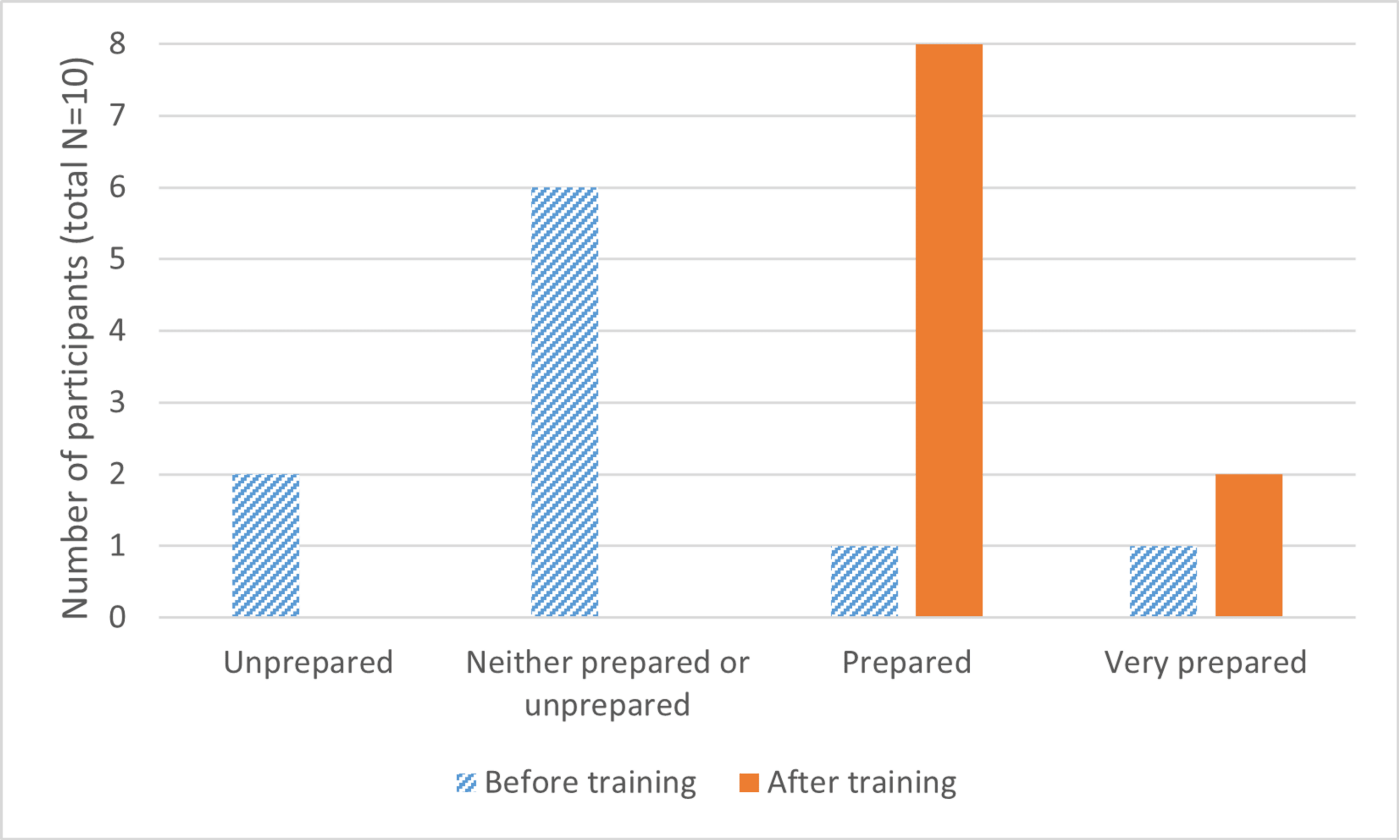
Responses to Likert-scale question asking participants how prepared they felt to break bad news to patients before and after engaging in conversation with the virtual patient.

Increased confidence in communicating with patients was also reflected by self-reported effectiveness, as shown in Figure 2. Only two participants indicated feeling “prepared” or “very prepared” to break bad news before engaging with the simulation; all 10 participants indicated one of these responses after completing the training module.

## Discussion

This study demonstrated the feasibility, acceptability, and self-reported effectiveness of a GPT-4-enabled conversational medical simulation module designed to help medical students practice empathetic discussions in difficult conversations with patients about abnormal mammogram findings suggesting the need for breast biopsy. Feasibility was emphasized by all 10 participants completing the study. Successful integration of the module into an easily accessible LMS suggested a scalable pathway to deploy simulations at the institution level beyond our pilot study. Acceptability and self-reported effectiveness were indicated by qualitative feedback and quantitative data, e.g., high SUS and TAM.

Our study reinforced findings from prior studies demonstrating the feasibility of GPT to support virtual communication skills training for clinicians and added new insights. Our study demonstrated the ability of GPT-4 to support verbal learner-VP communication for a more realistic interaction that maintained the high level of performance of prior LLM-focused studies that relied on text-based communication between learners [12,14,20]. Our simulation tested the ability of GPT-4 to handle complex and emotionally charged conversations around uncertain test results related to a possible cancer diagnosis. As noted earlier, most simulations have focused on more routine clinical encounters, with a notable exception where Webb20 tested the ability of ChatGPT-3.5 to train emergency room physicians to deliver the news of a serious diagnosis in the emergency room. Like our study, Webb [20] prompted ChatGPT to provide feedback to the learner based on the SPIKES protocol. A shared limitation in the feedback was that it was overly positive. In our study and Webb’s, SPIKES was a reasonable benchmark for providing feedback due to the “breaking bad news” nature of the conversation. In future iterations, we will consider more detailed prompts to improve feedback performance beyond the six categories of the SPIKES protocol. For example, Holderried et al. [14] developed a history-taking scenario and specified 42 categories that learners were to cover in their communication with the VP.

In addition to presenting a VP as a novel way to practice difficult conversations, our study contributes to the medical simulation literature by examining a complete communication skills training module, including initial modules that teach breaking bad news (didactic) and orient students to the simulation (pre-brief), VP conversation simulation (experiential learning), and concluding with written feedback. These components were adapted from traditional in-person SP communication training and are also crucial for effective VP training and communication skills retention. Likert-scale question responses (e.g., high SUS and TAM scores) and open-ended question responses were positive across all components of the training modules to reinforce the importance of a complete communication skills training module.

The breadth of possible training scenarios that students came up with for a communication skills training simulation like this reinforced acceptability to engage in VP training on an ongoing basis, possibly across different clinical rotations. Notably, students expressed an interest in training scenarios to practice discussions with patients for a range of diagnoses from mental health conditions to chronic illnesses like diabetes, and not just cancer. This highlights an important point about how conversations to break bad news are defined. As noted by one of our research team members (Lee) who is a primary care physician, physicians can become desensitized when diagnosing common illnesses that are life-changing for patients. Given the ability to program virtual simulations to cover conversations between providers and patients for a myriad of health conditions, virtual simulation may play an important role in preparing students for conversations with patients beyond what is feasible for in-person SP training. In addition to covering numerous health conditions, one student suggested a scenario with a VP and “multiple family members”. A scenario like this may be more viable through a virtual simulation that would not require multiple SPs to implement [30].

The main study limitation was that the pilot phase of development made it impractical to evaluate the most appropriate audience for the training module. Participants were a convenience sample of medical students in between their first and second years of medical education. It is possible that a training module like this may be more appropriate for students further along in their education, as indicated by comments from one participant. Future research should evaluate the timing as to when VP training should be offered to medical students, possibly serving different purposes depending on the participants’ year of medical education. The module could give first-year students a preview of likely conversations with patients as a primer for in-person simulation sessions and provide experienced students in later years a way to hone their communication skills for real clinical encounters. In addition, resident physicians could also benefit from utilizing more advanced communication training modules.

In a traditional simulation session, there would be a debriefing discussion with a facilitator after the simulation experience, and a two-way conversation would allow both the facilitator and learner to ask questions; facilitators could probe deeper into why a learner might have said something, or have the learner self-identify what they could have done better and then co-create a plan of action for change, instead of the AI feedback agent pointing what was said and providing alternate answers. Future iterations could incorporate a virtual coach that could engage the learner in a true “debriefing conversation” and provide feedback using effective debriefing techniques to further stimulate reflection and learning.

Our pilot study focused on feasibility and acceptability. Participants perceived being better prepared to break bad news to patients after communication with the VP. Future studies should evaluate whether self-perceived improvement translates to actual improvement in clinical communication skills with patients. This could be done by evaluating medical students’ improvements in Objective Structured Clinical Examinations focused on communication skills after exposure to the training module [31].

Due to the early stage of development, we also administered the module in-person at our institution’s medical simulation center, making it easier to troubleshoot potential technical issues during the pilot study. However, in-person training also restricted our ability to assess the ease with which medical students could remotely access the virtual simulation. Ease of remote access is a crucial factor for scalability, and further evaluation of our virtual simulation’s technical requirements and usability on older computers is warranted. As discussed earlier, we administered the module through Cornerstone, a web-based LMS. Although the pre-simulation and virtual simulation modules were completed in-person, all participants successfully completed the didactic module remotely. Future studies could explore the remote deployment of all modules to participants.

## Conclusions

This study demonstrated the feasibility, acceptability, and self-reported effectiveness of a GPT-4-enabled VP to help medical students practice discussing abnormal mammogram results with patients. The study leveraged GPT-4 advances to support a simulation involving more complex verbal physician-patient dialog than prior LLMs, and to provide automated qualitative feedback to learners. We recommend that future research evaluate when VP training should be offered to medical students in their coursework and how best to automate feedback mechanisms to improve learner performance and maximize VP training benefits. Research is also needed to evaluate the effectiveness of VP training using objective measures and return on investment to justify widespread adoption of these emerging technologies to supplement existing simulation modalities. Regardless of the mode of deployment, the designated audience, and other implementation aspects of virtual simulations, the growing number of VP studies and AI technology advances is creating momentum for a paradigm shift to incorporate VP-based training into medical education curricula.

## Appendices

Appendix A

**Table 4:** Questions for Qualtrics surveys filled out by participants before and after pilot testing the virtual patient simulation, including System Usability Scale (SUS; Q8-Q17) and Technology Acceptance Model questions (TAM; Q18-Q31).

Question number	Question	Response categories
Before simulation		
1	Have you ever received formal training on breaking bad news to patients?	(a) Yes; (b) No
1a	Specify (only asked if Q1 = 'Yes')	Text response
2	How prepared do you feel to break bad news to a patient?	(a) Very unprepared; (b) Unprepared; (c) Neither prepared or unprepared; (d) Prepared; (e) Very prepared
3	What is your experience with computer games (console, desktop and mobile)?	(a) Have little to no experience; (b) I'm a casual gamer; (c) I'm a hardcore gamer
4	How old are you?	Numeric response
5	What racial and ethnic categories do you identify with? (Check all that apply.)	(a) American Indian or Alaska Native; (b) Asian; (c) Black or African American; (d) Hispanic or Latino; (e) Middle Eastern or North African; (f) Native Hawaiian or Pacific Islander; (g) White; (h) Other
5a	Specify (only asked if Q5 = 'Other')	Text response
6	Please enter your sex assigned at birth.	(a) Male; (b) Female; (c) Other; (d) Prefer not to disclose
6a	Specify (only asked if Q6 = 'Other')	Text response
7	Please specify your gender identity (e.g., man, woman, nonbinary, agender).	Text response
After simulation		
8	Choose the number that matches how much you disagree or agree with each statement about the virtual training module that you pilot tested. Numbers range from 1 (I strongly disagree) to 5 (I strongly agree). I think that I would like to use this virtual training module frequently.	(1) I strongly disagree; (2) I disagree; (3) I do not agree or disagree; (4) I agree; (5) I strongly agree
9	I found the virtual training module unnecessarily complex.	(1) I strongly disagree; (2) I disagree; (3) I do not agree or disagree; (4) I agree; (5) I strongly agree
10	I thought the virtual training module was easy to use.	(1) I strongly disagree; (2) I disagree; (3) I do not agree or disagree; (4) I agree; (5) I strongly agree
11	I think that I would need the support of a technical person to be able to use the virtual training module.	(1) I strongly disagree; (2) I disagree; (3) I do not agree or disagree; (4) I agree; (5) I strongly agree
12	I found the various functions in the virtual training module were well integrated.	(1) I strongly disagree; (2) I disagree; (3) I do not agree or disagree; (4) I agree; (5) I strongly agree
13	I thought there was too much inconsistency in the virtual training module.	(1) I strongly disagree; (2) I disagree; (3) I do not agree or disagree; (4) I agree; (5) I strongly agree
14	I would imagine that most people would learn to use the virtual training module very quickly.	(1) I strongly disagree; (2) I disagree; (3) I do not agree or disagree; (4) I agree; (5) I strongly agree
15	I found the virtual training module very cumbersome to use.	(1) I strongly disagree; (2) I disagree; (3) I do not agree or disagree; (4) I agree; (5) I strongly agree
16	I felt very confident using the virtual training module.	(1) I strongly disagree; (2) I disagree; (3) I do not agree or disagree; (4) I agree; (5) I strongly agree
17	I needed to learn a lot of things before I could get going with the virtual training module.	(1) I strongly disagree; (2) I disagree; (3) I do not agree or disagree; (4) I agree; (5) I strongly agree
18	Instructions on how to use the virtual training module were clear and understandable.	(1) I strongly disagree; (2) I disagree; (3) I do not agree or disagree; (4) I agree; (5) I strongly agree
19	The user interface for the virtual training module was easy to navigate.	(1) I strongly disagree; (2) I disagree; (3) I do not agree or disagree; (4) I agree; (5) I strongly agree
20	I did not encounter technical issues (bugs) while using the virtual training module.	(1) I strongly disagree; (2) I disagree; (3) I do not agree or disagree; (4) I agree; (5) I strongly agree
21	The simulated phone call felt real to me.	(1) I strongly disagree; (2) I disagree; (3) I do not agree or disagree; (4) I agree; (5) I strongly agree
22	The simulated (AI-generated) patient responses seemed reasonable to me.	(1) I strongly disagree; (2) I disagree; (3) I do not agree or disagree; (4) I agree; (5) I strongly agree
23	I found the feedback I received at the end of the simulation to be helpful.	(1) I strongly disagree; (2) I disagree; (3) I do not agree or disagree; (4) I agree; (5) I strongly agree
24	Using the virtual training module would increase my confidence in communicating with patients.	(1) I strongly disagree; (2) I disagree; (3) I do not agree or disagree; (4) I agree; (5) I strongly agree
25	Using the virtual training module would improve my learning and performance in delivering difficult news.	(1) I strongly disagree; (2) I disagree; (3) I do not agree or disagree; (4) I agree; (5) I strongly agree
26	Using the virtual training module would make it easier for me to understand communication concepts.	(1) I strongly disagree; (2) I disagree; (3) I do not agree or disagree; (4) I agree; (5) I strongly agree
27	I enjoyed using the virtual training module.	(1) I strongly disagree; (2) I disagree; (3) I do not agree or disagree; (4) I agree; (5) I strongly agree
28	Using a virtual training module makes learning more interesting.	(1) I strongly disagree; (2) I disagree; (3) I do not agree or disagree; (4) I agree; (5) I strongly agree
29	I would like to use the virtual training module in the future if I have the opportunity.	(1) I strongly disagree; (2) I disagree; (3) I do not agree or disagree; (4) I agree; (5) I strongly agree
30	I would recommend the training module to others to help them practice difficult conversations with patients.	(1) I strongly disagree; (2) I disagree; (3) I do not agree or disagree; (4) I agree; (5) I strongly agree
31	I would like to see more of these types of virtual simulations for different types of patient encounters	(1) I strongly disagree; (2) I disagree; (3) I do not agree or disagree; (4) I agree; (5) I strongly agree
32	How prepared do you feel to break bad news to a patient?	(a) Very unprepared; (b) Unprepared; (c) Neither prepared or unprepared; (d) Prepared; (e) Very prepared
33	The last nine questions are open-ended questions for you to provide feedback on what you liked or found most useful and disliked or found least useful about the three sections of the virtual training module: (1) the introductory section containing instructions and background information you viewed on the screen immediately prior to the beginning of the simulated conversation with the patient; (2) the simulation where you practiced talking to the simulated patient; (3) and the feedback from your interaction with the simulated patient that was displayed on the computer screen. Please type responses in the text box following each question. What did you like or find most useful about the introductory section of the virtual training module?	Text response
34	What did you dislike or find least useful about the introductory section of the virtual training module?	Text response
35	What did you like or find most useful about the simulation section of the virtual training module?	Text response
36	What did you dislike or find least useful about the simulation section of the virtual training module?	Text response
37	What did you like or find most useful about the feedback you received through the virtual training module?	Text response
38	What did you dislike or find least useful about the feedback you received through the virtual training module?	Text response
39	What other topics or scenarios would you find appropriate for this type of virtual simulation training to help you gain experience and practice with being knowledgeable, humanistic and compassionate in delivering bad or difficult news to patients?	Text response
40	Besides virtual simulation training, what other formats would you find appropriate to help you gain experience and practice with being knowledgeable, humanistic and compassionate in delivering bad or difficult news to patients?	Text response
41	Please share any additional comments or suggestions.	Text response

Appendix B

**Table 5:** Text displayed on screen for five sections comprising the online didactic module. DM: diabetes mellitus; HTN: hypertension; COPD: chronic obstructive pulmonary disease; CHF: congestive heart failure

1. Introduction		
Delivering Difficult News to Patients		
One of the most difficult aspects of being a physician is delivering unfavorable news to patients about their health. While many communication guidelines exist, medical school training on this subject is limited. In interviews conducted with UCLA Health stakeholders (including primary care physicians, medical students, and breast cancer survivor patients), all interviewees identified a need for additional communication skills training for physicians around delivering bad or difficult news.		
“Looking back through my medical school and residency training… I never received any training on [delivering bad news].” - Primary care physician interviewee		
Why is Delivering Difficult News Important?		
While all interviewees identified the critical importance of difficult conversation training for physicians, many physicians are not well trained in this area, or do not feel competent in disclosing difficult information or diagnoses to patients. Reasons for this include: • Uncomfortable delivering difficult news (physicians are trained to help people, not hurt people); • Don’t know what to say; • Fear of not saying things well; • Fear of being blamed; • Fear of failure; • Fear of not knowing the answers. Effective delivery of challenging news to patients provides the following benefits:		
Prevents Litigation		
The delivery of challenging news is not only a fundamental aspect of patient care, but also a proactive measure to prevent potential legal disputes. Effective communication can prevent litigation from occurring, as many lawsuits stem from miscommunication between physicians and patients, rather than medical negligence.		
The corollary is that if medical negligence or malpractice does occur, your effective communication can make up for it and prevent a lawsuit from happening. Patients often sue doctors they hate. They rarely sue doctors they like.		
Diminishes Negative Emotions		
Effective communication also provides an opportunity to mitigate negative emotions such as anger and confusion. By ensuring that patients fully understand their situation, physicians can help alleviate these feelings and foster a more positive patient-doctor relationship.		
Helps Patients Through Difficult Situations		
Effective communication of difficult news can help patients navigate through potentially the most difficult time of their life or the worst news they have ever heard. Helping them through this situation empathetically and compassionately is an incredible talent on your part.		
Physician's Own Satisfaction		
When you learn how to deliver bad news well, it helps you feel more satisfied with your work and give you satisfaction that you did some good. Patient care can be exhausting and demoralizing, so it is important to gain satisfaction from successful empathic communication with patients during a trying time of their lives. This is one area you don’t want to fail in, because it can devastate a patient if done incorrectly.		
“But I don't need this lecture.”		
What if you're already a “good communicator and a very sympathetic person?” Answer: You still need this lecture. “Good” doctors who are “good” communicators and show sympathy are still sometimes ineffective at communicating bad news to patients. Properly communicating bad news is not as intuitive as one may think, but there is a method to the madness.		
2. History of Delivering Bad News to Patients in the US		
1951		
The Psychological Management of Cancer Cases		
In JAMA, 1951, Nathan Kline, MD of Worcester, Mass. and Julius Sobin, MD of Newark, NJ wrote an article entitled, The Psychological Management of Cancer Cases [32]. This article gave strategies of how to avoid telling a cancer diagnosis or how to psychologically deal with a patient once they find out they have cancer.		
Some of the tips include: • “Unless there are strong indications to the contrary, it is advisable to wait until the patient asks.” • “If the patient does ask, the way he phrases his question may give a strong indication as to the type of answer he desires. The ‘I don’t have cancer, do I?” phraseology is really only a request for negative reassurance and in all likelihood should be met with the tactics of evasion.” • “If it is found desirable to conceal from the patient the nature of his illness, it is usually wise to substitute some other condition.” • “A type of stomach ulcer, a kidney blockage, a liver dysfunction is the type of equivocal diagnosis suggested.” • “That a patient with this condition will dwell on it to some extent is to be expected. As previously outlined, efforts should be made to divert him from this type of preoccupation. In cases in which this is unsuccessful and the patient’s preoccupation becomes so severe that he makes life intolerable for both himself and his family, serious consideration should be given to one of the psychosurgical procedures. In addition to relieving intractable pain, prefrontal lobotomy usually results in the loss of this preoccupation with self and future, which leads some chronically ill patients to suffer out of all proportion to their illness. Other techniques, such as hypnosis and intensive psychotherapy, are valuable in some cases.”		
1961		
What to Tell Cancer Patients		
An article in JAMA in 1961 by Donald Oken, MD in Chicago, entitled What to Tell Cancer Patients revealed that 90% of physicians surveyed would prefer not to disclose a diagnosis of cancer to a patient [33].		
In this study, some of the physician responses for their reasoning were: “Most patients do not want to know,” “No one can be told without giving up and losing all hope,” “They always know anyway”, “Knowledge of cancer is ‘a death sentence,’ ‘a torture’”, “Telling is ‘the cruelest thing in the world,’” “awful,” and “hitting the patient with a baseball bat.”		
1977		
Change in Physician's Attitudes Toward Telling the Cancer Patient [34]		
The same questionnaire as the one used in the 1961 study was submitted to university hospital medical staff in 1977 to assess changes in attitudes. The results showed a complete reversal of attitude, with 97% of the 264 respondents indicating a preference for telling a cancer patient their diagnosis.		
Shift Toward Patient-Centered Care		
The shift towards the open disclosure of difficult news to patients in the US was a part of a wider shift in healthcare towards patient-centered care. Patient-centered care emphasizes the importance of patient involvement in healthcare decisions, respecting patient rights, ensuring transparency in medical error disclosure, and continuous improvement in treatment outcomes. It emphasizes the principles of patient autonomy, beneficence, and health justice.		
As a part of this shift, protocols and tools have been developed to aid physicians in the delivery of difficult news and diagnoses, while maintaining an empathetic communication style and a patient-centered focus.		
Further Reading on Breaking Bad News		
Title: How to Break Bad News: A Guide for Health Care Professionals; Author: Robert Buckman, MD; Year of Publication: 1992		
In this book, Dr. Robert Buckman of Toronto-Sunnybrook Regional Cancer Centre provides an overview of his six-step protocol for delivering bad news to patients with challenging diagnoses [22]. This protocol provides steps and tools physicians can use to help communicate difficult news to patients empathically and with clarity. Dr. Buckman's work has been foundational to the field of delivering difficult news in healthcare, and his six-step protocol was later developed into the oncology-specific SPIKES framework in 2000.		
Cultural Preferences for Bad News Delivery		
In many countries outside the US, not telling the truth in diagnosis of serious illnesses remains a common practice. Many patients come from cultures where it is customary for unfavorable news to be delivered to a family member, rather than directly to the patient. Los Angeles is a very cosmopolitan city, and we need to keep in mind the many different cultural backgrounds and cultural preferences of people in our community.		
3. SPIKES Protocol Overview		
The SPIKES protocol was developed by a team led by Dr. Walter F. Baile at the University of Texas, Houston and co-author Dr. Robert Buckman from the Toronto-Sunnybrook Regional Cancer Centre. It was created in response to the need for a more structured and systematic method of delivering information to patients about bad news or difficult diagnoses. The protocol is based in part on Dr. Buckman’s six-step strategy for communicating bad news, but is more oriented towards oncology. Nevertheless, the SPIKES protocol is applicable to many clinical settings and conditions, not just serious cancer diagnoses. This protocol is also helpful for disclosing chronic diseases such as DM, HTN, COPD. Some of the advice in the protocol will be helpful to improve your bedside manner, even if you are not giving bad news. SPIKES helps physicians and medical students approach giving bad news with a logical progression in mind, similar to the way they approach a procedure, reading a CXR, or working up a patient with CHF.		
SPIKES Acronym		
S = Setting; P = Perspective; I = Invitation; K = Knowledge; E = Emotion/Empathy; S = Strategy/Summary		
Steps of the SPIKES Protocol		
Learn each step of the SPIKES protocol for delivering difficult news to patients.		
S = Setting		
Get the setting right.		
• Deliver news in person: Being in person allows for real-time responses to patients’ emotional cues, showing the patient they are valued and you are committed to their care. SPIKES can also be followed in remote settings, although it may be harder to read emotional cues. • Presence of family/friends: Post-disclosure, patients may express anger at the physician. Having family or friends present can offer an objective perspective and act as a buffer, such as saying “I don’t think the doctor meant that.” • Introduce yourself to everyone: If appropriate, introduce yourself and shake hands with everyone in the meeting, shaking hands with the patient first. Establish each person’s relationship to the patient, and make each person feel important. If you do not acknowledge family or friends, they may work against you because you did not seem to care about them. • Private setting: Choose the most private setting possible, minimizing any distractions. If in a clinic, use a private room or office and turn off electronic devices. If a phone call, ensure minimal distractions and ask the patient to move to a private area. • Sit down: If in person, ensure that everyone is seated and you are facing the patient at eye level. This promotes better communication, builds rapport, and creates a safer environment for the patient. • Convey calmness, don't rush: Appear calm and unhurried. The manner of delivering bad news greatly impacts patient well-being. A rushed encounter could make patients feel anxious, neglected, or unheard.		
P = Perspective		
Ask the patient what they know.		
It is important to gauge what the patient knows about their own condition. Examples of a question to ask the patient are: • “Could I ask what your perspective is on what’s causing your symptoms?” • “What is your understanding of all that’s been happening?” • “What do you know about your illness so far?” Asking this type of question accomplishes 3 things: • It gives you the opportunity to ask a lead-in question so you don’t have to give the diagnosis right after walking in the door. • The patient will recognize that you are interested in what they think. • You will discover the patient’s level of understanding of their medical condition. Furthermore, asking these questions allows you to know what educational level you should relay information to the patient. You will also find out what type of emotional state the patient is in.		
I = Invitation		
Ask how much the patient wants to know and who should be told. It is very important to find out the patient's and family’s desires about disclosure. For example, many elderly patients would rather have their children told instead of them.		
Examples of a question you can ask are: • “Would you rather be told the results privately, or together with your family, or have me tell your family instead? Which do you prefer?” • “Would you like for me to tell you the full details of your condition? If not, is there somebody else you would like me to tell?” • “If your condition turns out to be something serious, whom should I give the results to?” (Best asked during the time of testing)		
The issue is, at what level does the patient want to know their diagnosis? Most patients will know if things are not going well. You must find out whether the patient wants the information disclosed overtly or not at that time.		
Cultural preferences regarding truth telling, cancer disclosure, and patient autonomy vary among patients, families, and physicians across the world. Physicians must recognize that people from various cultural backgrounds may not want to be told of their serious diagnoses (at that time), and we should always be cognizant of this.		
K = Knowledge		
Give the knowledge/diagnosis.		
Align your explanation with the patient’s understanding and acknowledge the patient’s own perspective of their symptoms. This gives the patient confidence in you because they will feel they have been heard and taken seriously. Using the patient’s own words, tell them the diagnosis in the most straightforward terms possible. Avoid technical jargon or euphemisms.		
For example: • “You’re right that breast cysts can cause these symptoms. But unfortunately, your biopsy came back as breast cancer.” • “I’m afraid the results are not what we hoped. The biopsy shows that you have colon cancer.” Note that after the diagnosis has been disclosed, most patients do not hear much of anything else, and are unlikely to remember finer details.		
Respond to the patient’s reactions empathetically.		
• Disbelief or denial: Physicians often respond to the patient’s disbelief by attempting to explain the patient’s misconception of the situation. Your response should rather be based on acknowledging how difficult it must be to receive the bad news. You might say something like, “It must be difficult to accept this, especially since you have been so healthy.” • Silence: It may be a manifestation of shock. Silence is uncomfortable for many physicians. Our impulse may be to start talking. One piece of advice is to remain silent until the patient begins speaking. If a large amount of time has elapsed, you may ask, “What are you thinking about right now?” • Anger or blame: Patients may blame or get angry at the messenger. Try to avoid arguing with them. Patients tend to decrease their anger when it meets a submissive response. Anger is often a sign that they are actually scared. • Crying: Lend supportive words to the patient. If the meeting is in person, offer the patient tissues.		
S = Strategy/Summary		
Appropriate treatment plan		
First, find out the patient's level of understanding of their diagnosis. The two most important questions to ask are: • “What do you know about your diagnosis?” (Lymphoma, RA, DM, breast cancer, HTN, COPD) • “Do you know anybody with the same illness?” You will be surprised at their answers, learn their level of understanding, and learn how to tailor their treatment.		
Some other guidelines: • Find out the patient’s concerns and questions: you may ask, “What are you expecting to happen?” • Identify and incorporate other sources of support, spiritual support, for example. • Check patient’s level of understanding: while explaining the treatment plan, verify with the patient that you are making sense. It may be helpful to put instructions in writing. • Limit amount of detail: Try not to say too much, because the patient won’t retain much of what you say after hearing the news. Don’t go into unnecessary details unless asked. • Convey uncertainty when appropriate: It’s okay to say to the patient, “I don’t know.” • Collective support: Say “we” will get you through this together, not just “you” will get through this. • Assess patient safety: Before the patient leaves, assess their safety and support at home. • Give hope: The key in your whole approach is you want to give the patient hope. Even if the prognosis is grim, do not rob patients of their hope. • Outline and summarize: To recap the meeting, briefly outline and summarize the diagnosis and the treatment plan. Two of the biggest concerns from patients after receiving bad news: • They feel left on an “island”, with a lack of support or lack of a good treatment plan. • They feel a loss of control. Thus, it’s important to let the patient know that: • There will be a team that will help them through their entire treatment course. • They will have a lot of say in their treatment plan. • They can message you anytime with questions or needs.		
Tailoring SPIKES		
The SPIKES protocol does not need to be followed every time a physician gives bad news. Physicians should tailor the steps of SPIKES for each individual and for each specific situation, just like we individualize general treatments guidelines for treating DM, HTN, or dyslipidemia.		
4. Communicating About Mammogram Results		
Mammograms are a key tool in the early detection of breast cancer, but interpreting results and communicating about them with patients can be challenging for physicians due to the inherent uncertainty of the results. Unlike delivering news of serious diagnoses, discussion of mammogram results must be more nuanced and should not typically be framed as bad news. Mammogram results themselves are not a definitive indication of cancer, and there is a significant risk of both false positives and false negatives. When communicating about mammogram results with patients, physicians must balance conveying the seriousness of any potential conditions versus the diagnostic uncertainty of mammograms.		
BI-RADS		
The American College of Radiology created the Breast Imaging Reporting and Data System (BI-RADS®) to categorize and rank mammogram images according to their risk of cancer. Review the following table to learn what each category of BI-RADS indicates.		
BI-RADS Category	Finding	What This Means
0	Incomplete	Additional imaging evaluation and/or comparison to prior mammograms is needed to assess whether the finding is abnormal.
1	Negative	No masses, distorted structures, or suspicious calcifications were found.
2	Benign finding	A benign finding is described, such as benign calcifications, masses, or lymph nodes in the breast.
3	Probably benign finding	The finding has a very low chance of being cancer (<2%). Follow-up with repeat imaging in 6 to 12 months is suggested.
4A	Suspicious abnormality (low suspicion of malignancy)	The chance of the finding being cancer is greater than 2% but less than 10%. A biopsy should be considered.
4B	Suspicious abnormality (medium suspicion of malignancy)	The chance of the finding being cancer is greater than 10% but less than 50%. A biopsy should be considered.
4C	Suspicious abnormality (high suspicion of malignancy)	The chance of the finding being cancer is greater than 50% but less than 95%. A biopsy should be considered.
5	Highly suggestive of malignancy	There is a high likelihood of cancer (>95%). A biopsy is strongly recommended.
6	Known biopsy-proven malignancy	There is a known breast cancer diagnosis. Appropriate action should be taken.
Source: Findings on a Mammogram and Mammogram Results - Komen.org [35]		
Addressing Abnormal Mammogram Results		
Discussing abnormal mammogram results with patients can be a challenging task for physicians. It often involves striking a delicate balance between providing the necessary information about the patient’s condition and managing the patient’s anxiety over the results. It’s important to inform patients that while an abnormal mammogram can be a cause for concern, it does not automatically mean the patient has cancer. In fact, most abnormalities detected on mammograms are not cancer.		
The conversation about mammogram results typically happens over a phone call, especially when the results do not indicate a definitive diagnosis of cancer. This allows for timely communication about the results while reducing the patient’s wait time and anxiety. Telehealth is also an option, offering a more personal touch compared to a phone call, while still maintaining the convenience of remote communication.		
On the other hand, a more serious diagnosis such as a biopsy-confirmed cancer diagnosis is typically discussed in person. This allows for a more comprehensive discussion, better emotional support, and immediate answers to any of the patient’s questions. It also provides an opportunity to discuss the next steps in detail, such as treatment options and follow-up care.		
Source: Getting Mammogram and Other Test Results - BreastCancer.org [36]		
Using the SPIKES Protocol to Discuss Abnormal Results		
Although a suspicious abnormality on a mammogram should not be framed as entirely bad news, the SPIKES protocol can still be useful for discussing challenging mammogram results with a patient.		
Here are some example statements following SPIKES that can be used by a physician during a patient phone call or video chat discussing concerning mammogram results.		
Setting		
When the setting is a phone call or video chat rather than in-person, it is important to ensure that you and the patient are both in a private place with minimal distractions.		
Example responses: • “Is now a good time to talk for you? Are you in a private place?” • “Could you please confirm that you’re in a comfortable and private place where we can discuss your mammogram results without interruptions?” • “I just want to let you know, I have disabled notifications and have turned off my pager so we will not be interrupted during our discussion.”		
Perspective		
Elicit the patient’s perspective on what they think their mammogram results mean.		
Example responses: • “Before we go into the details, could you please share with me your understanding of your mammogram results?” • “What is your interpretation of what you're seeing on the results?” • “I'd like to get your perspective on what you think these results mean.” • “How are you feeling about the results based on what you've seen so far?”		
Invitation		
Find out how much the patient would like to know about the results, and who should also be informed.		
Example responses: • “Could you please let me know how much detail you would like to hear about when discussing the results? Some people prefer a broad overview, while others want to know every detail.” • “How are you with medical knowledge? Would you like a detailed rundown of the mammogram results?” • “Would you like to have a family member or friend present for this discussion?”		
Knowledge		
Explain the results to the patient in straightforward terms, balancing the diagnostic uncertainty of mammograms with the possibility that the patient has breast cancer.		
Example responses: • “Your mammogram results are showing some areas where that are some suspicious masses. This doesn’t necessarily mean you have cancer, but further tests such as a biopsy will be needed to understand what these abnormalities are.” • “We found an area of concern on your left/right breast. It is not conclusive, but we have reason to suspect it could potentially be a more serious condition such as breast cancer. However, there are many other things it could be. Mammograms by themselves cannot confirm a diagnosis, which is why we suggest a biopsy procedure.” • “There is a pattern of growth on your left/right breast. While there is a risk that this growth could be something serious such as cancer, there is also a good chance it could be something less serious. It’s important to clarify that mammograms alone cannot provide a definitive diagnosis, which is why we recommend a biopsy procedure.” • “We are seeing a mass on your left/right breast. While a mass like this can be an indication of serious conditions like breast cancer, most abnormalities found on mammograms end up being benign, or non-cancerous. This is why we recommend a biopsy procedure, which will give us a clearer picture of what is really going on.”		
Emotion/Empathy		
Without the benefit of being in person and using body language to convey empathy, verbal communication of empathy becomes key. It is important to put yourself in the patient’s shoes to understand why they are fearful or anxious, or why they agree or disagree with your recommendations. Focused reassurance and a delicate balance of conveying seriousness and uncertainty will help the patient maintain hope regarding their future diagnosis.		
Example responses: • “I can imagine that this might be a lot to take in. How are you feeling at the moment?” • “I understand that seeing results like this can be very unsettling. It’s completely normal to feel this way. Many patients have a similar reaction to yours, but often find out after their biopsy that the abnormality was not serious after all.” • “So let’s stop right there, I know this is a lot to process. I’m curious to know what you’re feeling right now, and what is giving you the most concern?” • “It’s natural to feel anxious or worried about results like this. How are you coping with this news? Did you hear any good news in what I just said?” • “It can be a lot to deal with, and a biopsy certainly does have a scary connotation. But rest assured, we will help you through the process and support you, and I am always here to answer any and all questions you might have. You can call me anytime to discuss.” • “Do you have any reservations about the biopsy procedure we are recommending? I’d be happy to explain the specifics of the procedure in more detail to help ease your concerns.”		
Strategy/Summary		
The patient may express concerns about the biopsy procedure. It is important to respond to the patient’s concerns empathetically, explain the justification for a biopsy, and provide a plan of action. The physician should wrap things up by summarizing all of the points covered in the meeting.		
Example phrases: • “Our recommendation is that you schedule a biopsy appointment to get to the bottom of what these results mean. This will allow us to send a tissue sample in for testing to check for the presence of any cancerous cells.” • “Here’s what we could do next. We could schedule your biopsy to get a better understanding of these abnormalities. After we have those results, we would reconvene. How does that sound to you?” • “To recap, the results of your screening mammogram are showing a concerning area of growth on your left/right breast. While it could be something serious like cancer, there’s also a good chance it is non-cancerous. Further testing via a biopsy will be necessary to determine a definitive diagnosis, and we would like to schedule this appointment as soon as you’re ready. Do you have any questions for me before we wrap up?”		
Communicating Diagnostic Uncertainty		
Diagnostic uncertainty is inherent in mammography. Here are some tips on how to communicate this uncertainty: • Acknowledge the uncertainty: Be honest about the limitations of mammograms and the possibility of false positives and false negatives. • Emphasize the precautionary principle: Explain that further tests, like a biopsy, are sometimes needed to ensure nothing is missed, even if the likelihood of cancer is low. • Reassure the patient: Reassure the patient that diagnostic uncertainty is common across all of healthcare.		
It’s important to note that mammograms correctly identify about 87% of women who have breast cancer. The chance of having a false positive result after one mammogram ranges from 7% to 12%. After 10 yearly mammograms, the chance of having at least one false positive result is about 50-60%. Conversely, the chance of having a false negative result after one mammogram is around 20%.		
Source: Getting Mammogram and Other Test Results - BreastCancer.org [36], Mammography - Komen.org [37]		
Providing Explanation of Biopsy Procedure		
If a patient asks why they need a biopsy or expresses fear about a biopsy, there are several ways you could provide a simple explanation that will clarify what the procedure entails and address the patient's concerns.		
• Explain the purpose of a biopsy: “In a biopsy, we extract a small sample of tissue. This sample will be sent to a lab where it will be tested for the presence of cancerous cells. This gives us a lot higher accuracy than a mammogram can by itself.”		
• Clarify why a biopsy is needed: “A biopsy is usually recommended if we see an abnormality that has some risk of being cancer, but it does not mean that what you have is cancer. A mammogram alone is not a confirmation of anything; it’s a tool we can use to identify suspicious areas that warrant further testing. A biopsy will reveal the root cause of any masses we are seeing on the mammogram.”		
• Discuss the procedure and address safety concerns: “During a biopsy, we numb the area of your breast, and a small sample of tissue is collected using a small needle. This sample is then sent to a lab for further analysis. The biopsy will be guided by imaging such as ultrasound, and a small metallic marker may be placed at the biopsy site for future reference.”		
• Address concerns about pain and recovery: “Most patients describe the sensation during a biopsy as pressure rather than pain. Local anesthesia will be applied, which will numb the area and eliminate the need for putting you to sleep. The risk of infection is low, and recovery time is usually short, but it can vary depending on the amount of bleeding or bruising.”		
• Address patient preference for getting another mammogram instead: “While imaging tests like CT scans or MRIs are helpful in detecting masses or irregular tissue, they alone cannot tell the difference between cancerous cells and non-cancerous cells. A biopsy, on the other hand, can determine with greater certainty whether a tumor or growth is cancerous or non-cancerous.”		
5. Delivering Bad News Conclusion		
Communicating unfavorable news is a critical responsibility for physicians, and poses many unique challenges. Especially when delivering news of serious diagnoses, the manner in which the news was delivered and the empathy communicated by the physician can have a tremendous impact on the mental and emotional well being of patients and families. Structured protocols for delivering bad news such as the SPIKES protocol offer physicians useful guidelines to ensure that these critical discussions are always approached with sensitivity, compassion, and professionalism. Protocols such as SPIKES can also be useful beyond the context of serious or grave diagnoses. In the case of screening mammograms, the results of a scan are often inconclusive, and there is a significant risk of false positive and false negative results. Patients who have an abnormal or suspicious mammogram result may be particularly anxious and afraid about the implications of their results. The SPIKES protocol can provide physicians with a systematic patient-centered communication method that asks for the patient’s perspective on the results, explains clearly and succinctly what those results mean, and is responsive to the patient’s emotional reactions.		
When it comes to giving bad news: Patients may never forgive you if you do it badly. But they may never forget you if you do it well.		
Putting Difficult Conversation Skills into Practice		
Now that you have a foundation in some tools and techniques that can be employed when breaking bad or difficult news, you can put these important skills into practice.		
In our next training, you will complete a virtual simulation of a difficult phone call to discuss a patient’s abnormal mammogram results. This scenario lets you rehearse an emotional phone conversation in a repeatable and risk-free environment, using a virtual patient driven by artificial intelligence. Patient responses are dynamically generated in real time, allowing you to practice following the SPIKES protocol, communicating diagnostic uncertainty, conveying empathy, building rapport with the patient, and more.		
